# Mechanisms of programmed cell death in livestock follicular development and atresia: a review

**DOI:** 10.1186/s40104-025-01241-6

**Published:** 2025-08-03

**Authors:** Shunshun Han, Yimeng Wei, Yuanhang Wei, Xiyu Zhao, Yuqi Chen, Can Cui, Yao Zhang, Huadong Yin

**Affiliations:** 1 Key Laboratory of Livestock and Poultry Multi-Omics, Ministry of Agriculture and Rural Affairs, College of Animal Science and Technology, Sichuan Agricultural University, Chengdu, 611130 Sichuan China; 2 Farm Animal Genetic Resources Exploration and Innovation Key Laboratory of Sichuan Province, Sichuan Agricultural University, Chengdu, 611130 Sichuan China

**Keywords:** Follicular atresia, Follicular development, Livestock reproduction, Programmed cell death

## Abstract

Programmed cell death (PCD), including autophagy, apoptosis, and ferroptosis, is a fundamental biological process that plays a critical role in follicular development and atresia in livestock. In ovaries, the vast majority of follicles undergo atresia, while only a small fraction reach ovulation. Emerging evidence suggests that these three forms of PCD are intricately involved in regulating follicular fate through distinct yet interconnected molecular mechanisms. This review summarizes recent advances in understanding the roles of autophagy, apoptosis, and ferroptosis in follicular development and atresia, with a focus on their molecular mechanisms and interactions. By elucidating the complex regulatory networks of PCD in ovarian physiology, this review aims to provide new insights into improving reproductive efficiency in livestock through targeted modulation of these pathways.

## Introduction

Follicular atresia is a physiological process of selective follicular degeneration in the ovary, serving as a crucial mechanism for maintaining ovarian function and reproductive health. During the female reproductive cycle, only a minority of follicles develop to maturity and ovulate, while the vast majority are eliminated through atresia [1]. This process not only ensures the selection of high-quality follicles but also maintains ovarian homeostasis by eliminating abnormal follicles. Programmed cell death (PCD) plays a central role in follicular atresia, primarily comprising apoptosis, autophagy, and ferroptosis. These modes of cell death regulate follicular fate through distinct molecular mechanisms and exhibit complex interplay, collectively influencing the progression of follicular atresia [2].

In recent years, with the in-depth investigation of PCD (autophagy, apoptosis, ferroptosis) mechanisms, their roles in follicular atresia have gradually been elucidated. Moreover, the interaction among different types of PCD holds significant importance in follicular atresia. For instance, autophagy can inhibit apoptosis by clearing damaged mitochondria to maintain cell survival [3], but under certain conditions, it may instead promote apoptosis [4]. Ferroptosis also exhibits complex regulatory relationships with autophagy and apoptosis, collectively influencing follicular fate [5]. Emerging evidence suggests that these three forms of PCD are intricately involved in regulating follicular fate through distinct yet interconnected molecular mechanisms. Targeted modulation of these pathways, such as CRISPR-Cas9-mediated knockout of pro-apoptotic *Bax* or upregulation of autophagy-related *ATG7* in granulosa cells [6], has shown promise in enhancing follicular survival and ovulation rates in bovine and porcine models, highlighting their translational potential for improving reproductive outcomes in livestock. This review summarizes recent advances in understanding the roles of autophagy, apoptosis, and ferroptosis in follicular development and atresia, with a focus on their molecular mechanisms and interactions.

## Physiological mechanisms of follicular development and atresia

### Follicular development: from primordial to mature follicles

**Fig. 1:**
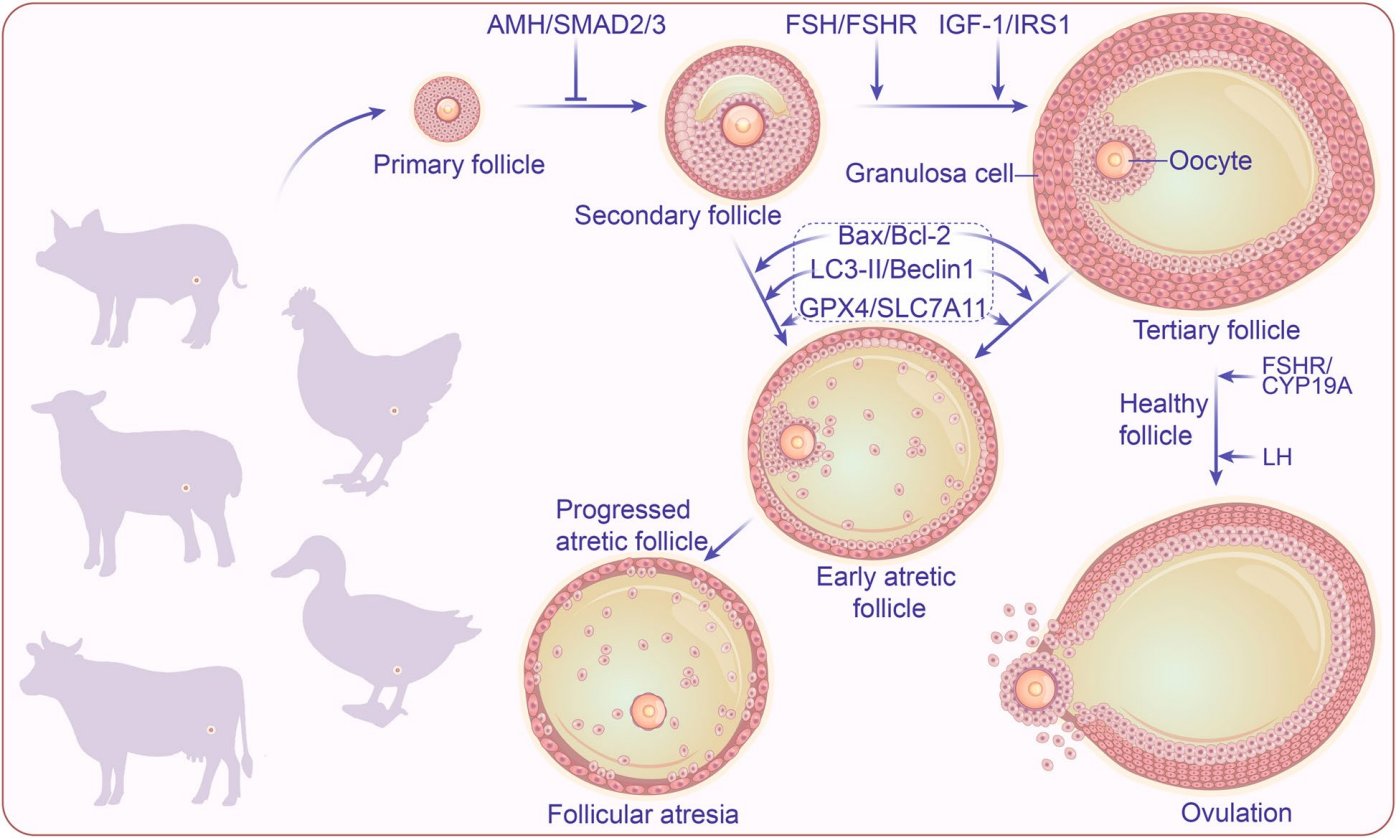
Schematic overview of follicular development and atretic progression. The developmental continuum commences with primordial follicles (single layer of granulosa cells), progresses through intermediate stages (stratified granulosa layers, no antrum), and culminates in antral follicles (fluid-filled antrum). While most follicles undergo atretic regression via PCD, a select subset achieve preovulatory maturation and ovulation, ensuring ovarian reserve renewal. Mammalian follicular development: Primary follicle → Secondary follicle → Tertiary follicle → Ovulation → Corpus luteum; Avian follicular development: Primary follicle → Secondary follicle → Tertiary follicle → Ovulation (no corpus luteum)

Follicular development and atresia are critical components of ovarian physiology, involving complex hormonal regulation and molecular mechanisms (Fig. 1). Follicular development begins with primordial follicles, which consist of oocytes surrounded by a single layer of flattened granulosa cells (GCs) and are maintained in a quiescent state through anti-Müllerian hormone (AMH) signaling [7]. AMH, secreted by GCs, suppresses primordial follicle activation via SMAD2/3-dependent pathways, ensuring a gradual recruitment of follicles into the growing pool [8]. Transition to primary follicles is initiated by follicle-stimulating hormone (FSH) binding to its receptor (FSHR) on GCs, which activates the PI3K/AKT/mTOR pathway to promote GC proliferation and differentiation [9]. Concurrently, insulin-like growth factor 1 (IGF-1) synergizes with FSH by phosphorylating IRS-1, further enhancing PI3K/AKT signaling and upregulating aromatase (CYP19A1) for estrogen synthesis [10]. In porcine models, as follicles progress to the secondary stage, transforming growth factor-beta (TGF-β) superfamily members, including growth differentiation factor 9 (GDF-9) and bone morphogenetic proteins (BMPs), play pivotal roles. GDF-9, secreted by oocytes, activates SMAD2/3 in GCs to promote hyaluronan synthesis and gap junction formation (e.g., connexin 37), facilitating oocyte-GC communication and nutrient exchange [11, 12]. Simultaneously, BMPs from theca cells inhibit FSH-induced progesterone production via SMAD1/5/8, ensuring proper follicular maturation and preventing premature luteinization [13].

In antral follicles, luteinizing hormone (LH) triggers theca cell androgen synthesis through upregulation of steroidogenic acute regulatory protein (STAR) and CYP17A1, while GCs convert androgens to estrogen via FSH-stimulated aromatase activity [14, 15]. The selection of the dominant follicle is mediated by a negative feedback loop involving peptide hormones: Activin**,** a TGF-β family member, enhances FSH sensitivity in GCs by upregulating FSHR expression, thereby promoting estrogen production and follicle survival [16]. However, rising estrogen levels suppress pituitary FSH release, depriving subordinate follicles of survival signals and leading to their atresia [17]. Additionally, inhibin B**,** produced by GCs of the dominant follicle, further suppresses FSH secretion, consolidating follicular dominance [18]. Other peptide regulators include KIT ligand from GCs, which binds KIT receptors on oocytes to maintain meiotic arrest via cAMP/PKA signaling, and neuropeptide Y**,** which modulates follicular angiogenesis through vascular endothelial growth factor (VEGF) regulation [19]. During ovulation, oxytocin secreted by luteal cells promotes follicular rupture by stimulating prostaglandin synthesis, highlighting the multifaceted roles of peptides in coordinating follicular development and ovarian function [20].

### Follicular atresia: mechanisms and regulation

Follicular atresia refers to the degenerative process by which ovarian follicles that do not reach full maturation undergo PCD and resorption [2, 21]. This process is a crucial component of ovarian function, ensuring that only a limited number of follicles ovulate during an animal’s reproductive cycle. The regulation of follicular atresia involves a complex interplay of hormonal, cellular, and molecular mechanisms: ① Endocrine Regulation: Key hormones such as FSH [22], LH [23], and AMH [24] modulate follicular growth and survival. A decline in FSH levels often initiates atresia in subordinate follicles. FSH promotes granulosa cell proliferation and inhibits apoptosis, while LH primarily regulates theca cell function and steroidogenesis during later stages of follicular development [25]. AMH, produced by granulosa cells of preantral and small antral follicles, plays a critical role in inhibiting primordial follicle recruitment and FSH-dependent follicular growth, thereby preventing premature follicle depletion and maintaining ovarian reserve [26]. ② Intra-ovarian Factors: Local growth factors (e.g., insulin-like growth factors, transforming growth factor-beta) and cytokines contribute to follicle selection and apoptosis [27–29]. ③ Cellular Pathways: PCD in GCs, triggered by mitochondrial dysfunction, oxidative stress, and activation of caspase pathways, is the hallmark of atresia [30]. ④ Epigenetic and Non-coding RNAs: Recent research highlights the role of miRNAs and circRNAs in post-transcriptional regulation of genes involved in follicular apoptosis and survival [31, 32].

Follicular development and atresia are a dynamic equilibrium process. Under normal circumstances, atresia is a physiological phenomenon that helps maintain the proper function of the ovaries [33]. However, abnormal atresia can lead to ovarian dysfunction, such as premature ovarian failure and decreased fertility [34, 35]. For livestock breeding, a deep understanding of the mechanisms of follicular development and atresia provides a theoretical basis for improving reproductive performance and offers new targets and strategies for molecular breeding to further meet the demand for efficient reproduction in modern animal husbandry.

## Fundamental mechanisms of programmed cell death

Apoptosis, autophagy, and ferroptosis are among the most extensively studied forms of PCD and differ significantly in their morphological and molecular characteristics (Table 1). Apoptosis is of great significance for maintaining homeostasis and individual development, participating in physiological activities such as embryonic development [36] and immune system homeostasis [37]. Apoptosis progresses through early (cell shrinkage, endoplasmic reticulum expansion) [38], middle (chromatin condensation, nuclear membrane rupture) [39], and late (formation of apoptotic bodies that are engulfed) [40] stages. Apoptosis is closely related to activation of caspases, a class of proteolytic enzymes that belong to the cysteine protease family and play a crucial role in homeostasis and PCD [41]. Apoptosis operates through multiple mechanisms, such as the formation of DISC (death-inducing signaling complex) by the binding of FasL to Fas receptors, activating downstream caspases [42].

**Table 1:** A comparison of autophagy, apoptosis, and ferroptosis

Regulated cell death	Ferroptosis	Apoptosis	Autophagy
Key feature	Mitochondrial cristae reduction (disappearance); Outer membrane ruptured and shriveled; Mitochondria deeply stained	Chromatin aggregation and disruption; Nucleolus disappeared; Nuclear pycnosis and fragmentation	Autophagosome formation; Autophagy lysosome formation
Other feature	Iron-dependent preservation of nuclear integrity; Cell membrane rupture	Cell shrinkage; Cytoplasmic leakage and membrane vacuolation	Nuclear changes; Cell membrane unchanged
Detection index	ROS, PTGS2 increased and NADPH decreased	Mitochondrial cytochrome c release; Caspase activation cascade; Intracellular Ca^2+^ overload	Conversion from LC3-I to LC3-II
Positive regulatory factor	Erastin, RSL3, RAS, Sorafenib, p53	p53, Bax, Bak, TGF-B, Dexamethasone, radiation	ATG family, Beclin1
Negative regulatory factor	GPX4, FTH1, FSP1, SLC7A11, NRF2, Ferrostatin-1, Liproxstatin-1, DFO	Bc1-2, Bcd-XL, Z-VAD-FMK, IL-4	mTOR, 3-Methyladenine, Wortmannin, Spautin-1

Autophagy mediates follicle growth and development by clearing damaged organelles and misfolded or aggregated proteins, thereby maintaining cellular homeostasis [43]. Depending on the pathway by which cellular materials are transported to lysosomes, autophagy is divided into macroautophagy, microautophagy, and chaperone-mediated autophagy [44]. The mammalian target of rapamycin (mTOR) signaling pathway plays a critical role in the initiation, progression, and termination of autophagy through precise mechanisms [45]. Besides, the mTOR-independent pathways (AMPK, PI3K, Ras-MAPK, p53, PTEN, endoplasmic reticulum stress) are also extensively involved in autophagy [46].

Ferroptosis is a novel form of PCD that is iron-dependent and distinct from apoptosis, and autophagy [47]. It is characterized by the accumulation of intracellular iron ions and the exacerbation of lipid peroxidation, ultimately leading to cell death [48]. The mechanisms of ferroptosis mainly include iron metabolism imbalance, abnormal fatty acid metabolism, glutathione metabolism blockade, the action of other enzymes, the role of signaling pathways, and the transduction regulation of related signaling pathways [49]. Elevated levels of intracellular iron ions are a key feature of ferroptosis. Iron ions promote the production of reactive oxygen species (ROS) through the Fenton reaction, particularly hydroxyl radicals, thereby triggering lipid peroxidation [50]. In addition, the peroxidation of polyunsaturated fatty acids (PUFAs) in the cell membrane can disrupt the integrity and function of the cell membrane [51]. Lipid peroxidation products, such as malondialdehyde (MDA), increase in content [52].

## Crosstalk among PCD pathways

Different types of PCD are intricately interconnected and collaboratively regulate the process of follicular atresia. These forms of PCD interact at the molecular and cellular levels, creating a regulatory network that determines the fate of ovarian follicles.

### Autophagy and apoptosis

**Fig. 2:**
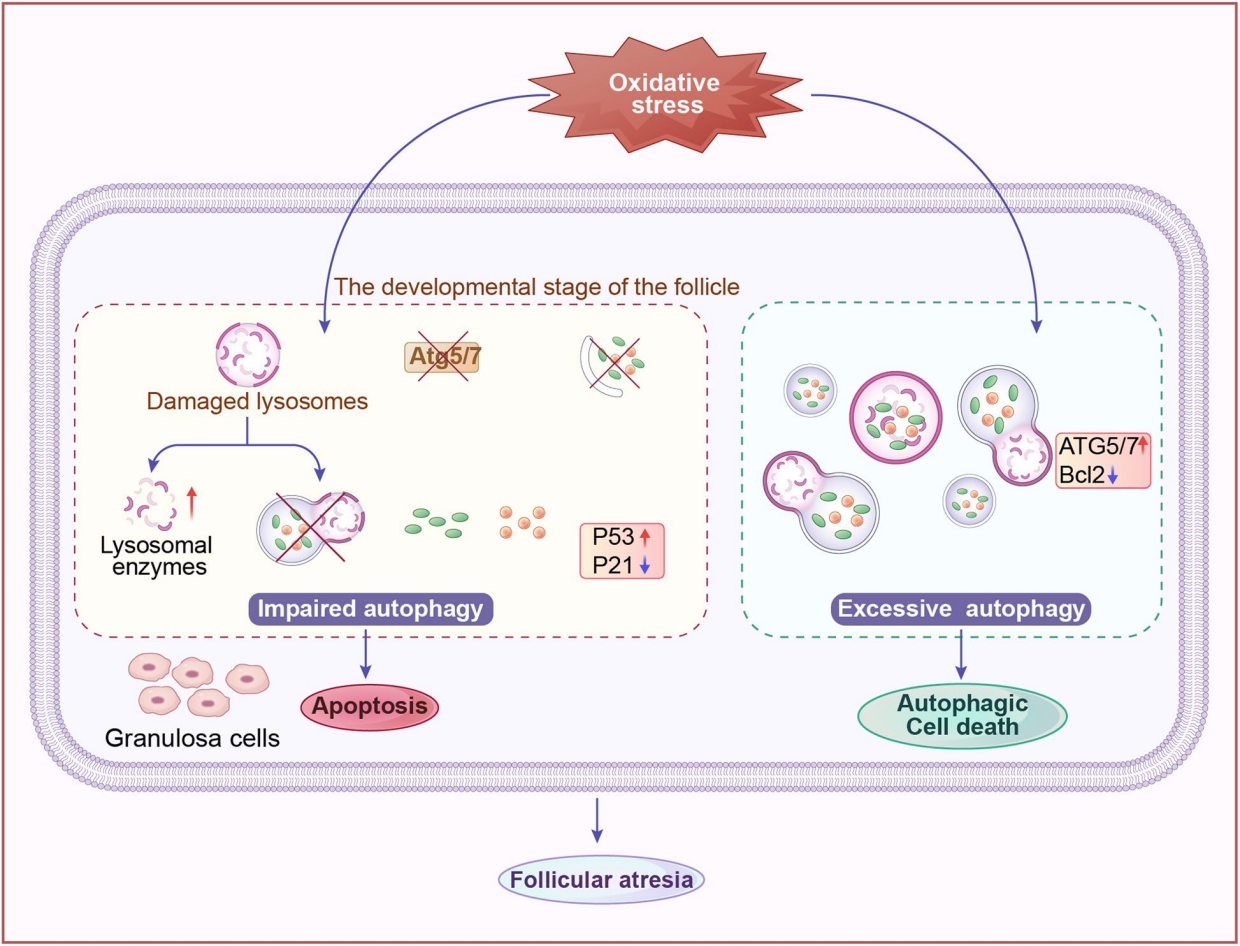
Interplay between autophagy and apoptosis in follicular atresia. Genetic ablation of autophagy genes (*ATG5*/*ATG7*) disrupts autophagic flux, leading to compensatory apoptosis activation, premature senescence, and pro-inflammatory cytokine secretion. Oxidative stress induces lysosomal membrane permeabilization, releasing hydrolases, while excessive autophagy triggers autophagic cell death. These pathways synergistically drive granulosa cell demise and atresia progression

Autophagy and apoptosis are closely intertwined, with autophagy often serving as a protective mechanism to maintain cellular homeostasis. However, under certain conditions, autophagy can also promote apoptosis [53]. For instance, moderate autophagy can degrade damaged organelles and proteins, thereby the activation of signaling pathways [54]. Conversely, excessive autophagy can lead to autophagic cell death, which shares many features with apoptosis [46]. This interplay is particularly evident during follicular atresia, where the balance between autophagy and apoptosis determines the fate of follicular cells [55]. During the early stages of follicular atresia, moderate autophagy can degrade substances related to the production of ROS within the cell, reducing ROS-induced cellular damage and, consequently, decreasing the release of apoptosis-inducing factors and inhibiting the activation of apoptotic signaling pathways [56, 57]. However, excessive autophagy may cause lysosomal rupture, releasing hydrolases and other substances that activate the caspase family, initiating the apoptotic program [58]. Therefore, the interplay between autophagy and apoptosis is critically involved in the regulation of follicular development (Fig. 2).

### Autophagy and ferroptosis

Autophagy also interacts with ferroptosis, another form of PCD that is characterized by iron-dependent lipid peroxidation [59]. Autophagy can mitigate ferroptosis by removing damaged mitochondria and reducing the production of ROS, which are key drivers of ferroptosis [60]. However, under conditions of severe cellular stress, autophagy can exacerbate ferroptosis by promoting the accumulation of iron and lipids [61]. This dual role of autophagy highlights its complex regulation in follicular atresia.

**Fig. 3:**
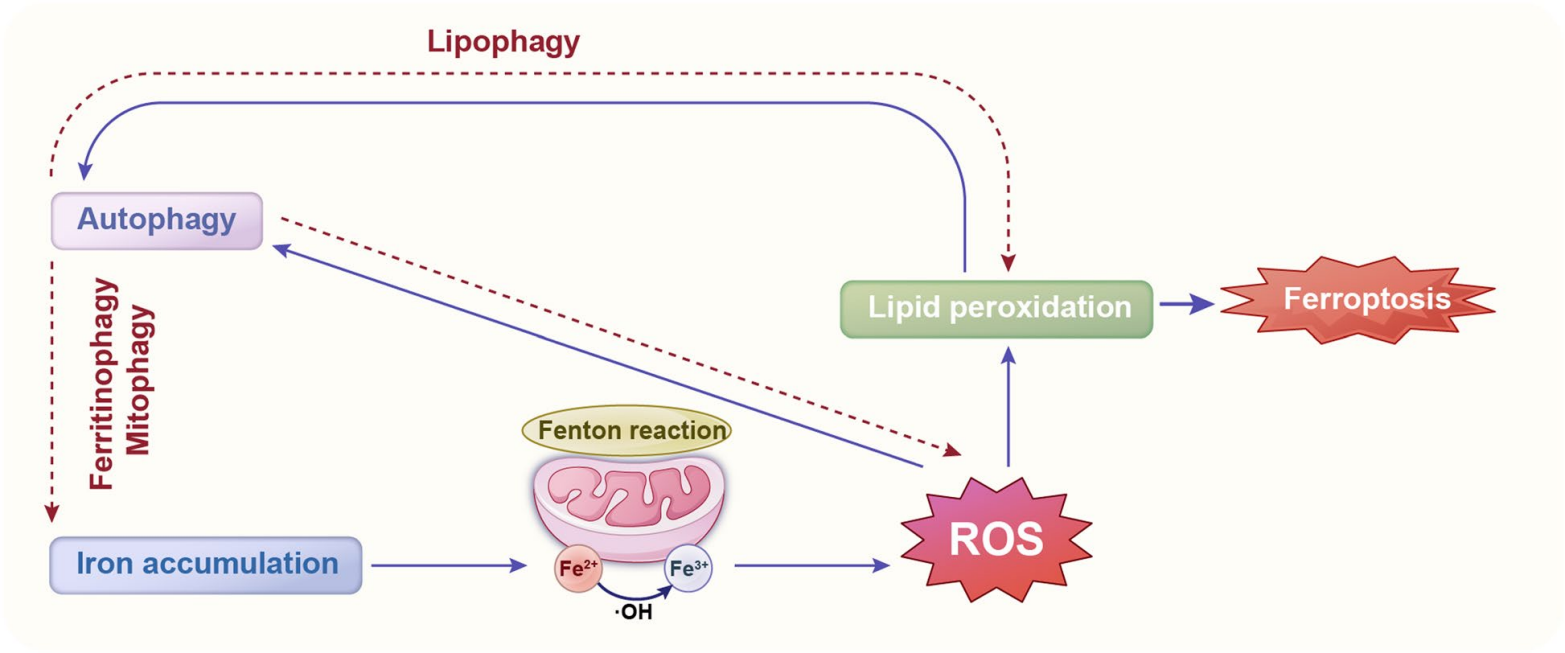
Regulatory role of autophagy in ferroptosis via iron-dependent and lipid peroxidation pathways. Ferroptosis initiation involves reactive oxygen species (ROS) generation from iron overload or lipid peroxidation, activating autophagy that amplifies labile iron pool expansion and ROS production via a positive feedback loop. Notably, ferroptosis occurs independently of autophagy under iron-mediated ROS elevation, whereas autophagy alone fails to induce ferroptosis without ROS. Autophagy thus acts as an amplifier, not an essential component, of ferroptosis

During ferroptosis, lipid peroxidation and other damages can activate cellular stress responses, thereby inducing the occurrence of autophagy [62]. Additionally, autophagy can clear damaged mitochondria and other organelles [63], reducing the production of ROS [64], which is one of the key factors inducing ferroptosis. Therefore, autophagy can inhibit ferroptosis to a certain extent. Apoptosis-related proteins, such as the Bcl-2 family, can influence the generation and release of ROS by regulating mitochondrial membrane permeability, thereby inhibiting ferroptosis [65]. The interplay between autophagy and ferroptosis critically regulates cell death through synergistic or antagonistic interactions (Fig. 3).

### Apoptosis and ferroptosis

Apoptosis and ferroptosis can also influence each other. Apoptosis-related proteins, such as the Bcl-2 family, can regulate the generation and release of ROS, thereby modulating ferroptosis [66]. Additionally, ferroptosis can induce apoptosis by causing extensive lipid peroxidation and membrane damage, leading to the activation of apoptotic signaling pathways [67]. This cross-talk between apoptosis and ferroptosis underscores the intricate regulatory networks that govern follicular atresia [68].

Since ROS is one of the key factors inducing ferroptosis, the effective control of ROS by the apoptotic pathway suppresses the activation of ferroptosis-related signaling pathways to a certain extent, and cells preferentially choose apoptosis as the mode to progress towards cell death. Conversely, the overload of ROS and lipid peroxidation products generated during ferroptosis can activate apoptotic signaling pathways, making the cell more susceptible to apoptosis [69].

The interplay between autophagy, apoptosis, and ferroptosis creates a complex regulatory network that determines the fate of ovarian follicles [70]. During follicular development, these pathways work in concert to maintain follicular homeostasis and select high-quality follicles for ovulation. However, during atresia, the balance between these pathways shifts, leading to the elimination of non-viable follicles [71]. Understanding this integrated regulation is essential for developing targeted interventions to improve reproductive efficiency in livestock.

### Programmed cell death across follicular developmental stages

During the early stages of follicular development, PCD helps in the selection and maturation of follicles, ensuring that only the most viable ones proceed to ovulation [72]. In contrast, during the later stages, PCD can lead to atresia if the follicles fail to meet the necessary criteria for maturation [30].

During this stage, PCD is minimal, and the primary focus is on maintaining the quiescent state of the follicles [73]. As follicles progress to the primary stage, PCD becomes more active, primarily through autophagy, which helps in removing damaged organelles and proteins, ensuring the proper development of the follicle [74]. In the secondary follicle stage, the role of PCD shifts towards apoptosis, which helps in selecting the most viable follicles for further development [75]. The presence of high-quality follicles is ensured by eliminating those with compromised oocytes or GCs. This stage is also marked by increased hormonal regulation, with FSH promoting the proliferation and differentiation of GCs [76]. As follicles mature into tertiary and dominant follicles, the balance between PCD and follicular survival becomes critical. Apoptosis continues to play a role in eliminating excess GCs and oocytes, while autophagy and ferroptosis may contribute to the regulation of cellular stress and lipid metabolism [77]. The dominant follicle, which is selected for ovulation, undergoes a complex interplay of PCD mechanisms to ensure its readiness for ovulation. Understanding the distinct roles of PCD in each follicular stage is essential for elucidating the molecular mechanisms underlying follicular development and atresia. This knowledge can provide new targets for improving reproductive efficiency in livestock through targeted modulation of these pathways.

## Role of autophagy in follicular development and atresia

### Autophagy in follicular development: protective or destructive?

**Fig. 4:**
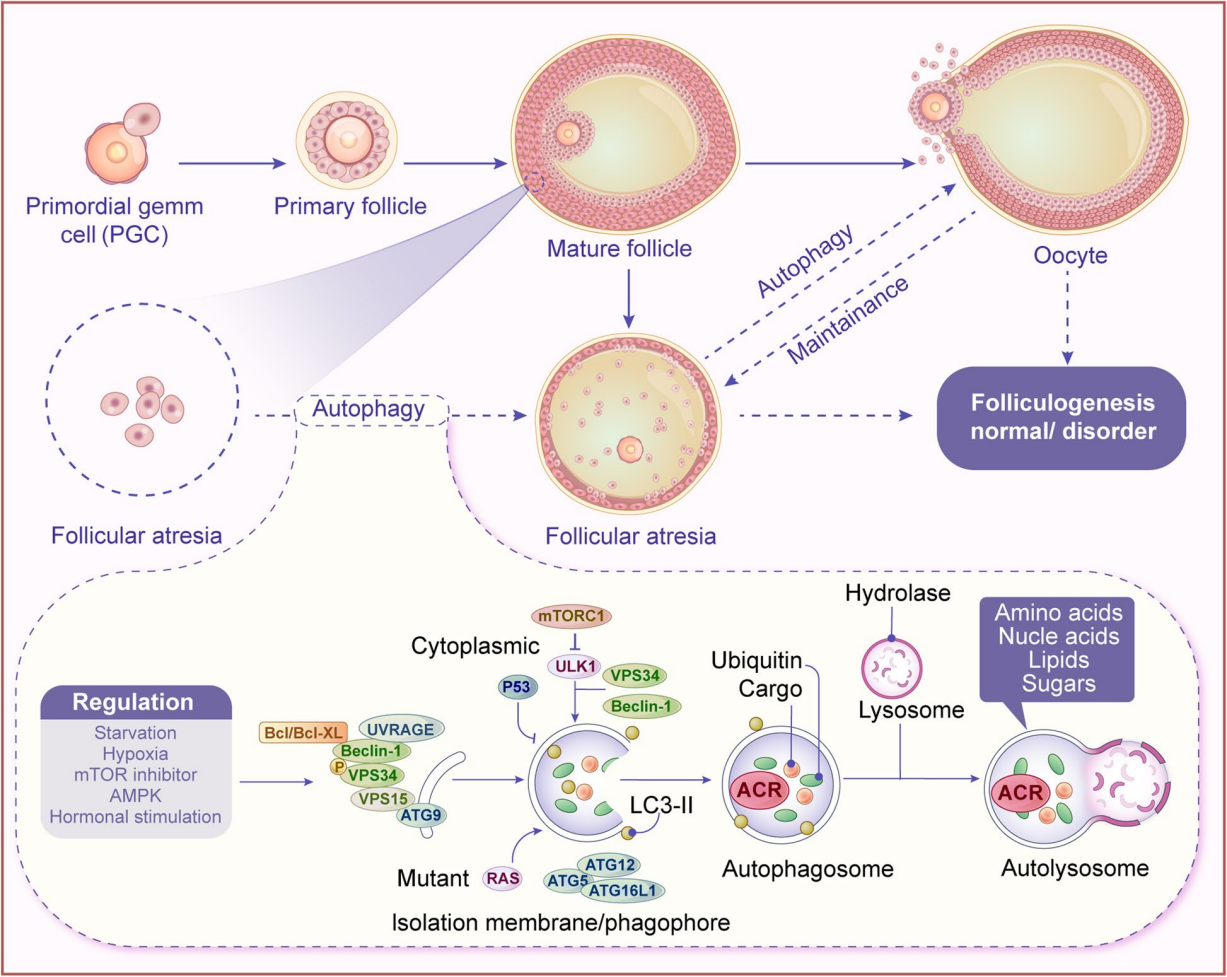
Dual regulatory roles of autophagy in follicular homeostasis. Autophagy maintains follicular cell homeostasis by degrading damaged organelles and proteins, ensuring cellular function. During development, moderate autophagy removes impaired components, supporting granulosa cell and oocyte survival. However, excessive autophagy under oxidative stress triggers autophagic cell death, contributing to atresia. ATG5/ATG7-mediated autophagy regulates this process. Autophagy modulation balances follicular development and atresia, critical for reproductive health. Thus, autophagy dual regulates follicular survival and atresia

Follicular development is a highly regulated process involving the interaction of cells within the follicle, oocytes, and GCs [74]. Autophagy directly influences follicular development and its ultimate fate of atresia by regulating energy balance, protein degradation, apoptosis, and stress responses [78]. In the early stages of follicular development, autophagy supports oocyte health and survival by clearing damaged components and dysfunctional proteins, maintaining an optimal cytoplasmic environment. Furthermore, GCs within the follicle also utilize autophagy to remove damaged organelles, sustaining their metabolic and growth functions, which are essential for proper follicular development (Fig. 4). However, in the later stages, excessive or dysregulated autophagy can lead to follicular atresia in chicken [79]. Overactive autophagy accelerates the degeneration of follicular cells, especially in cases where ovulation fails, a phenomenon commonly seen in various pathological conditions like polycystic ovary syndrome (PCOS) [80]. Studies indicate that the mTOR signaling pathway plays a critical role in follicular development and atresia, with mTOR modulating autophagy by preventing its overactivation. In particular, mTOR inhibition can enhance autophagic activity, facilitating the clearance of follicular cells and eventually leading to follicular atresia [33]. Additionally, *AMPK*, a key upstream regulator of autophagy, is involved in energy regulation during follicular development. Energy imbalance and abnormal AMPK activity are closely associated with follicular atresia and ovarian aging. Research suggests that upregulation of AMPK promotes autophagy in the follicle, enhancing the oocyte's ability to adapt to stress while inhibiting follicular atresia [81]. Therefore, autophagy plays a dual role in follicular development and atresia, reflecting the complex regulation of ovarian physiology, energy metabolism, and cellular health. Understanding the precise regulation of autophagy in follicular development and atresia is vital for advancing research on ovarian diseases, assisted reproductive technologies, and ovarian aging [74].

### Autophagy-related genes and their expression in follicular atresia

Autophagy-related genes show differential expression during follicular atresia in various animal models. The expression of *ATG7*, a key gene in autophagosome formation, remains constant throughout oocyte development stages, and *ATG7*-deficient mice exhibit significant reproductive defects, underscoring its critical role in germ cell survival [82, 83]. Furthermore, LC3 is expressed in GCs at various stages of follicular development and in pre-antral follicles, suggesting a correlation between autophagy and granulosa cell-mediated follicular atresia [82]. Additionally, Beclin-1, another key protein involved in autophagy initiation, is widely expressed in follicular cells during the late stages of atresia [84].

In poultry, the nesting behavior is closely linked to follicular atresia, with GC autophagy playing a pivotal role. Lou et al. [85] observed that autophagy in GCs during the nesting period of geese was associated with increased ROS levels, which activate the mTOR signaling pathway, thereby modulating autophagy and contributing to nesting-induced follicular atresia. This finding is consistent with another studies, where the expression of autophagy-related genes such as *LC3*, *ATG12*, Beclin1, *p53*, and *p62* was significantly higher in the ovaries of geese during the nesting period than during the laying period [86]. This suggests that oxidative stress-induced GC autophagy is a major factor in follicular atresia. Further studies by Jiang et al. [87] using a 3-NPA-induced oxidative stress model in geese demonstrated that spermidine, a natural autophagy inducer, significantly upregulated the LC3-II/I ratio, suppressed p62 expression, and reduced ROS levels and apoptosis in GCs, inhibiting follicular atresia. Liu et al. [88] discovered that PHB2 combined with ERβ induces autophagy in PGCs by targeting the mTOR pathway, thereby affecting the atresia of porcine ovarian GCs. Moreover, various coding genes and non-coding RNAs play important regulatory roles in the autophagic process of follicular atresia.

For instance, the circadian rhythm gene *NR1D1* affects the autophagic process in GCs by regulating the expression of the autophagy-related gene 5 (*ATG5*), thus influencing the progression of follicular atresia. Han et al. [89] found that autophagy induced by the silencing of fibromodulin may be a cause of follicular atresia in chicken. In terms of non-coding RNAs, He et al. revealed a miRNA regulatory network in chicken follicular atresia through miRNA sequencing. Further cellular experiments demonstrated that miR-30a-5p can directly target and inhibit the expression of Beclin1, thereby suppressing autophagy in GCs [90]. miR-21-3p inhibits autophagy in bovine GCs and induces apoptosis of bovine follicular granulosa cells by targeting *VEGFA* and suppressing the PI3K/AKT signaling pathway, thereby facilitating the process of follicular atresia [91]. A study by Lv et al. [92] identified miR-221-5p_R-4 as a regulator of internalized trehalose-dependent autophagy through its interaction with NRBF2 in porcine GCs. Furthermore, He et al. [93] identified a differentially expressed circRNA, *RALGPS2*, between normal and atretic follicles. This circRNA encodes a novel protein circRALGPS2-212aa that can promote chicken GC autophagy by forming a protein complex with PARP1 and HMGB1 [93].

### Hormonal regulation of autophagy in granulosa cells

Reproductive hormones play a critical role in the process of follicular development and atresia, especially in follicular growth, maturation, and selective atresia. The regulation of these processes by hormones is essential [94]. Increasing evidence suggests that the regulatory effects of these hormones are mediated through autophagy [95]. Liu et al. [96] found that FSH can promote progesterone synthesis and secretion in porcine GCs. This stimulatory effect is achieved through the PI3K/JNK/c-Jun pathway, which promotes autophagy, accelerates the degradation of lipid droplets, and increases the expression of steroidogenesis-related genes. Another study in pigs demonstrated that FSH can also activate mitochondrial autophagy in porcine GCs via the HIF-1α-PINK1-Parkin cascade, thus preventing cell apoptosis caused by hypoxia [97]. Additionally, research by Han et al. [98] on yaks discovered that miR-23a can enhance GC autophagy by targeting and inhibiting the ASK1/JNK axis. This also increased apoptosis, the abundance of estrogen receptor α/β, and the secretion of estradiol and progesterone. FSH and LH can activate these biological processes by increasing the levels of miR-23a. Similarly, Tang et al. found that high doses of FSH promote autophagy in bovine GCs by directly targeting the AKT/mTOR signaling pathway [99]. Interestingly, Liu et al. demonstrated that ERβ can also activate autophagy through the AKT/mTOR signaling pathway, suggesting that estrogen may also be involved in the regulation of bovine GC autophagy [100]. In addition, some non-reproductive hormones can regulate autophagy in follicular GCs. For example, adiponectin promotes GC autophagy in geese, preventing apoptosis and follicular atresia caused by oxidative stress [101]. Lecot-Connan et al. discovered that AMH not only protects the ovarian reserve by inhibiting the growth of primordial follicles but also sustains their survival through the activation of autophagy [102]. Furthermore, hormones such as melatonin and antioxidants like vitamin E can alleviate oxidative damage-induced autophagy in murine GCs by inhibiting the signaling pathways of SIRT1, FoxO1, JNK/Bcl2/Beclin1 [103]. While the hormonal regulation of autophagy in follicular cells has been increasingly studied, the potential feedback mechanisms whereby autophagy may influence hormone receptor expression remain largely unexplored. In particular, whether autophagic activity modulates FSHR or LHR expression to impact follicular fate warrants investigation. Recent studies in other cell systems have demonstrated that autophagy can regulate membrane receptor turnover and downstream signaling cascades [104, 105]. Given the critical role of gonadotropin receptors in follicular survival, future studies should examine if autophagy-mediated receptor degradation represents a novel pathway contributing to follicular atresia. This potential bidirectional crosstalk between PCD pathways and hormonal signaling could provide important insights into follicular selection mechanisms.

## Role of apoptosis in follicular development and atresia

### Granulosa cell apoptosis: a key driver of follicular atresia

**Fig. 5:**
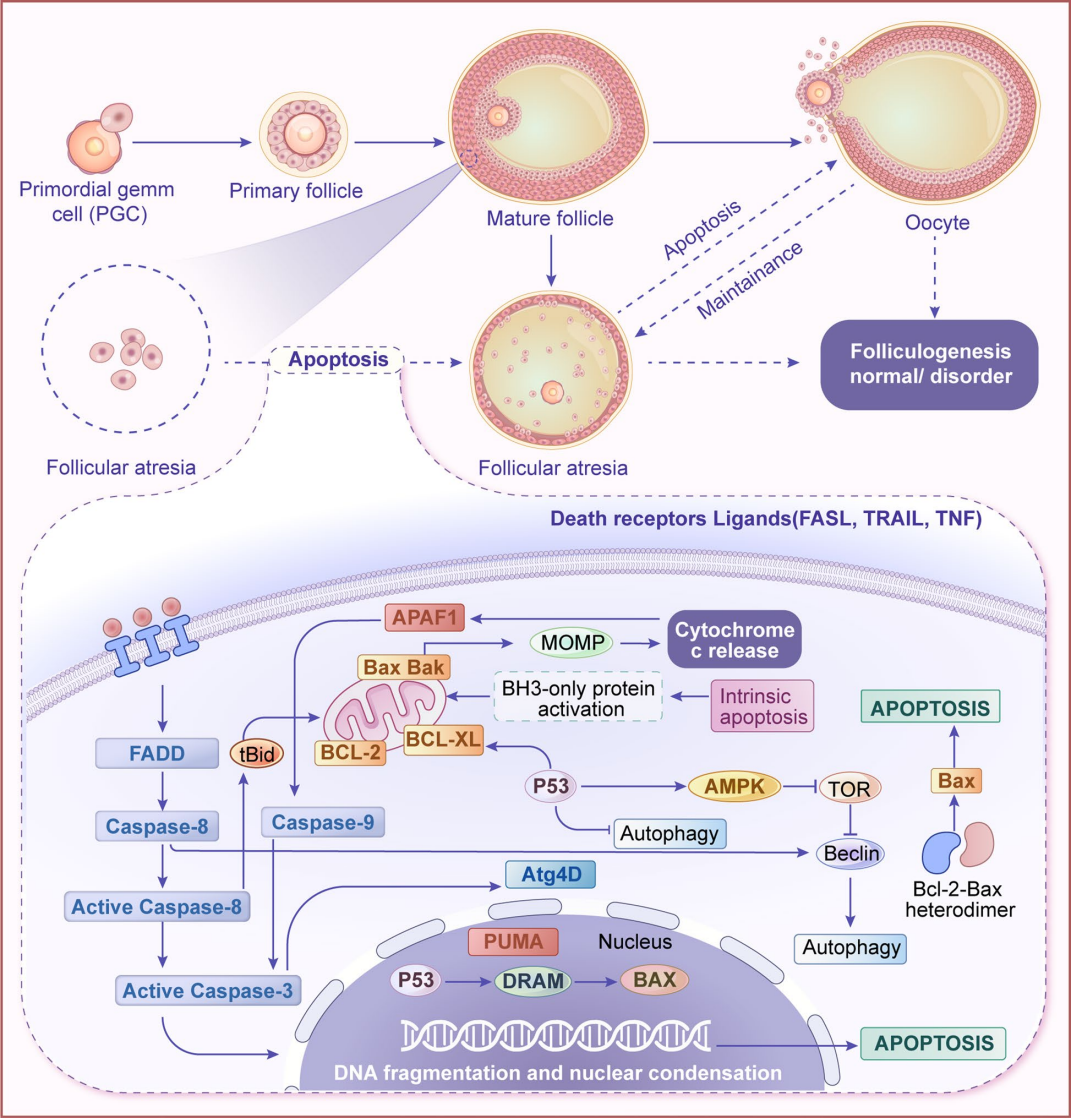
Apoptotic regulation of follicular quality control. Apoptosis preserves follicular quality by eliminating abnormal cells during development. Oxidative stress or damage activates apoptotic pathways, inducing programmed cell death in granulosa cells and oocytes, thereby initiating atresia. This process is regulated by Bcl-2 family proteins and caspase cascades. Moderate apoptosis maintains follicular homeostasis and prevents defective follicle accumulation, whereas excessive apoptosis accelerates atresia, causing granulosa cell loss, impaired follicular development, and compromised reproductive health

Apoptosis, a tightly regulated PCD mechanism, is characterized by the controlled degradation and fragmentation of cellular proteins and genomic DNA [106]. Apoptosis, a fundamental biological process integral to both developmental stages and aging, serves as a pivotal biological mechanism that orchestrates tissue morphogenesis, maintains organismal homeostasis, eliminates compromised or stressed cells, and modulates immune system functionality [107]. In response to cellular damage induced by pathogenic conditions or toxic insults, apoptosis is activated as a protective homeostatic mechanism to eliminate compromised cells and maintain tissue integrity [108]. Apoptosis serves as a central mechanism underlying follicular atresia in the reproductive system (Fig. 5). Precise regulation of GC apoptosis within ovarian follicles exerts profound impacts on follicular development and subsequent reproductive competence, highlighting its therapeutic potential for managing fertility-related disorders.

Substantial evidence has established apoptosis as a critical determinant in follicular atresia. During this degenerative process, GC apoptosis and associated alterations in the follicular microenvironment emerge as pivotal factors influencing ovarian function. In a seminal work by Tilly et al., chicken GC apoptosis was first identified in avian follicles, with their findings demonstrating that follicular atresia is triggered when granulosa cell apoptosis exceeds 10% in developing follicles [109]. Follicular atresia is mediated through a sequential apoptotic cascade. Johnson et al. demonstrated in avian models that the initiation of follicular regression originates from apoptotic events in GCs, subsequently extending to the theca cell layer in hen. This physiological process is precisely regulated by a multi-level apoptotic signaling network encompassing both pro-apoptotic and anti-apoptotic factors [110]. Liu et al. found that the environmental toxin microcystin-LR induces ovarian cell apoptosis in mice through transcriptional upregulation of DNA damage-inducible transcript 3, thereby accelerating follicular atresia [111]. Studies on sheep GCs reveal that targeted induction of apoptosis in these somatic cells directly contributes to folliculogenesis impairment and accelerated follicular atresia, through mechanisms involving dysregulation of pro-survival signaling pathways [112]. Endoplasmic reticulum stress-induced apoptosis of GCs has been demonstrated to trigger follicular atresia in zebrafish ovarian follicles [113].

In addition, elucidating the molecular mechanisms underlying granulosa cell apoptosis provides crucial insights for developing targeted anti-apoptotic therapies. Such therapeutic advancements could effectively preserve ovarian reserve function by preventing pathological follicular atresia, while simultaneously mitigating risks associated with aberrant follicular cell proliferation and subsequent neoplastic transformation [114, 115]. Furthermore, investigating the apoptotic mechanisms of GCs during follicular atresia may provide critical insights into mitigating ovarian tissue inflammation and fibrosis [116]. Oocyte quality exerts a direct impact on critical reproductive parameters including fertilization rate and ovarian reserve maintenance. Notably, apoptosis serves as a key pathological mechanism that significantly compromises oocyte developmental competence, ultimately leading to follicular atresia. In bovine follicular studies, Zeuner et al. elucidated that apoptotic processes not only diminish oocyte developmental potential but also impair cellular quality through dual mechanisms: exacerbation of oxidative stress and disruption of mitochondrial functional integrity in bovine [117]. Apoptosis of oocytes serves as a key mechanism underlying the depletion of germ cells in the ovarian reserve [118]. Bildik et al. demonstrated that pharmacologically induced apoptosis in human oocytes leads to oocyte depletion, thereby initiating follicular atresia through germ cell insufficiency [119]. Heat stress-induced ceramide-mediated apoptosis has been demonstrated to impair oocyte maturation in porcine models [120]. Modulating programmed apoptosis can optimize follicular utilization efficiency, enhance oocyte developmental competence, and elevate fertilization rates, thereby significantly improving reproductive biotechnology outcomes [121]. Furthermore, the apoptosis of theca interstitial cells demonstrates significant correlation with follicular development. In a pioneering study, Zerbinatti et al. revealed that apolipoprotein E in murine ovaries participates in folliculogenesis regulation through dual mechanisms: modulating androgen production in theca cells and constraining the thecal cell population [122]. In experimental models of sepsis-induced ovarian pathology, Taşdemir et al. reported significantly increased apoptotic activity in theca interna cells of rats with compromised reproductive function [123]. In a pivotal study, Luo et al. demonstrated that targeted inhibition of theca-interstitial cell apoptosis significantly enhanced ovarian folliculogenesis and endocrine function in rat models [124].

### Related apoptosis genes and their expression in follicular atresia

The Bcl-2 protein family is the core of mitochondrial apoptosis regulation [125]. During follicular atresia, Bax upregulation induces mitochondrial outer membrane permeabilization, releasing cytochrome c (Cyt c) to activate caspase cascade via apoptosome assembly [126]. Emerging evidence indicates that combined pharmacological suppression of Bcl-xL and Bax activation synergistically reverses apoptosis resistance across malignancies, significantly enhancing tumor cell vulnerability [127]. Following apoptosis inhibition in porcine ovarian GCs, a marked upregulation was observed in the expression level of the anti-apoptotic *Bcl-2* gene, accompanied by a concurrent reduction in the expression of the pro-apoptotic Bax protein [128]. In the ovarian injury rat model, GCs exhibited a concomitant upregulation of pro-apoptotic Bax protein and downregulation of anti-apoptotic Bcl-2 expression [129].

As a key executor in apoptotic pathways, the caspase protease family plays a pivotal role in PCD. Notably, caspase-3 activation represents an irreversible biochemical commitment to apoptotic progression, marking the point of no return in cellular demise [106]. The transcriptional upregulation of another gene in the caspase family, caspase-8, shows a significant response to cellular stress and programmed death signals, especially during follicular degeneration [130]. Caspase-9 exhibits progressive upregulation during follicular atresia, with markedly elevated expression under pathological conditions linked to oxidative stress and mitochondrial dysfunction [131, 132]. Emerging studies have shown that caspase-3 mediates apoptosis in porcine GCs and regulates follicular dynamics and ovarian homeostasis during porcine reproduction [133]. Furthermore, caspase-3 serves as a key mediator of apoptotic signaling in human ovarian GCs, demonstrating its conserved regulatory role in PCD pathways [134]. Other studies have demonstrated a marked upregulation of caspase-3, caspase-8, and caspase-9 expression in GCs undergoing apoptosis in murine models [135].

As a pivotal tumor suppressor, p53 is markedly upregulated during cellular stress and critically drives granulosa cell apoptosis in follicular atresia by transcriptionally activating pro-apoptotic mediators like Bax and Puma, positioning it as a central regulator of follicular regression [136, 137]. Emerging evidence reveals that p53-ASPP1 interaction synergistically enhances p53's pro-apoptotic activity via coordinated nuclear translocation, exacerbating cardiomyocyte apoptosis in myocardial I/R injury [138]. Emerging evidence has revealed that the p53/SIRT1 signaling axis in human granulosa COV434 cells is modulated by TOPK, thereby effectively suppressing TNF-α-induced apoptotic processes in these ovarian follicular cells [139].

In follicular atresia, the Fas/FasL system critically regulates apoptosis. Recent studies in PCOS rat models reveal that the Fas/FasL/Caspase-8 axis critically regulates follicular atresia via apoptotic mechanisms [140]. Emerging evidence highlights apoptosis-associated genes, particularly *FOXO3* and *GDF-9*, as critical regulators of follicular development and atresia. Studies demonstrate that FOXO3 activation induces granulosa cell apoptosis through precise molecular mechanisms, driving follicular atresia [141]. Recent studies in human and murine GCs indicate that FOXO3 critically regulates apoptotic cascade initiation, thereby promoting follicular atresia [142]. As a* TGF-β* superfamily member, *GDF-9* critically regulates granulosa cell survival by activating the Akt/FOXO3a pro-survival signaling axis, as demonstrated by Monte et al. through phosphorylation-dependent inactivation of this apoptotic pathway [143].

These studies elucidate critical regulatory genes and signaling pathways governing granulosa cell apoptosis during follicular atresia, advancing molecular understanding of ovarian follicle regression. These mechanistic insights provide a framework for exploring ovarian pathophysiology and reproductive endocrine disorders, while identifying potential therapeutic targets for ovarian dysfunction and apoptosis-resistant malignancies.

### Hormonal regulation of granulosa cell apoptosis

Reproductive hormones including FSH, LH, estradiol (E2), and progesterone (P4) exert regulatory control over granulosa cell survival and apoptotic processes through multiple signaling pathways. During follicular development and atresia, these endocrine factors not only directly modulate granulosa cell proliferation and differentiation, but also orchestrate PCD by regulating the expression of apoptosis-associated genes [144].

FSH, a pituitary glycoprotein hormone and gonadotropin, acts via binding to FSH receptors on GCs. Early studies showed that FSH inhibits ovarian follicular apoptosis by suppressing DNA fragmentation, a hallmark of PCD [145]. In a recent study, Zhang et al. identified a novel FSH-mediated regulatory axis in mice where follicular fluid glutamine metabolism governs follicular homeostasis, directing ovarian development and ovulation [9]. Furthermore, FSH regulates GCs multifunctionally, primarily via the PI3K/AKT pathway, stimulating sex steroid biosynthesis/secretion while inhibiting apoptotic pathways, thereby maintaining cellular homeostasis and facilitating follicular development [146, 147]. FSH protects porcine ovarian GCs from hypoxia-induced apoptosis by activating the HIF-1α/PINK1/Parkin signaling axis, which enhances mitophagy to preserve cellular homeostasis under hypoxic stress [97]. Murine studies demonstrate that ovarian FSH receptor ablation disrupts follicular development, markedly reducing follicle counts during primordial follicle pool establishment and maintenance [148].

LH serves as a critical endocrine regulator during the mid-to-late stages of follicular development, playing indispensable roles in key biological processes such as follicular luteinization and ovulation induction [149]. Rossi et al. revealed that LH protects the primordial follicle reserve in prepubertal ovaries by counteracting cisplatin-triggered apoptotic mechanisms [150]. Emerging evidence suggests that LH-regulated apoptosis in rat premature ovarian failure models is mediated through intracellular oxidative stress modulation [151]. Longo et al. demonstrated that LH modulates early follicular development and acts as a key synchronizer for coordinated primary follicle growth [152].

Estradiol, a key steroid hormone in folliculogenesis, orchestrates granulosa cell homeostasis by coordinately regulating survival maintenance, mitotic progression, and phenotypic differentiation [153]. Murine studies demonstrate estradiol protects GCs from oxidative stress via dual mechanisms: suppressing ROS-mediated damage while upregulating antioxidant enzymes, thereby reducing apoptosis [154]. Progesterone, a pivotal post-ovulatory steroid hormone predominantly secreted by the corpus luteum, exhibits multifaceted regulatory effects during follicular atresia [155]. Further studies in human GCs have demonstrated that sustained progesterone exposure exerts anti-apoptotic effects on these somatic cells [156].

Reproductive hormones coordinate granulosa cell fate via complex signaling pathways, critically governing follicular development, ovulation, and atresia. Deciphering gonadotropin-mediated regulation of granulosa cell apoptosis reveals fundamental mechanisms of follicular destiny determination and reproductive senescence. Systematic exploration of apoptotic gene networks under endocrine control advances understanding of physiological folliculogenesis while exposing molecular drivers of ovarian aging. These insights enable novel therapeutic strategies through targeted modulation of hormone signaling or precision intervention in apoptotic regulators, offering potential solutions for ovarian dysfunction and age-related fertility preservation.

## Role of ferroptosis in follicular development and atresia

### Ferroptosis: mechanisms and role in follicular atresia

Ferroptosis is implicated in diverse diseases, including cancer, neurodegenerative disorders, and ischemia–reperfusion injury. Emerging but limited evidence suggests its potential role in ovarian physiology, where iron overload in atretic follicles correlates with granulosa cell ferroptosis, hinting at a possible mechanistic link to follicular development and atresia [157]. However, these findings remain largely observational, and causal relationships require further validation, particularly in vivo. Iron is indispensable for ovarian function, participating in steroidogenesis and follicular maturation. Both iron deficiency and overload impair follicular development. Iron deficiency disrupts Hippo/YAP signaling, hindering secondary-to-antral follicle transition [158, 159]. Conversely, iron overload in GCs may induce ferroptosis via ROS overproduction, destabilizing HIF-1α and FSHR expression [160, 161]. Chen et al. demonstrated that Fe^2^^+^ accumulation in GCs significantly enhances reactive oxygen species (ROS) production via Fenton reactions, which could lead to suppressed GC proliferation and impaired follicular development [162]. However, whether these mechanisms directly translate to follicular atresia in vivo, especially in livestock species, remains unclear.

**Fig. 6:**
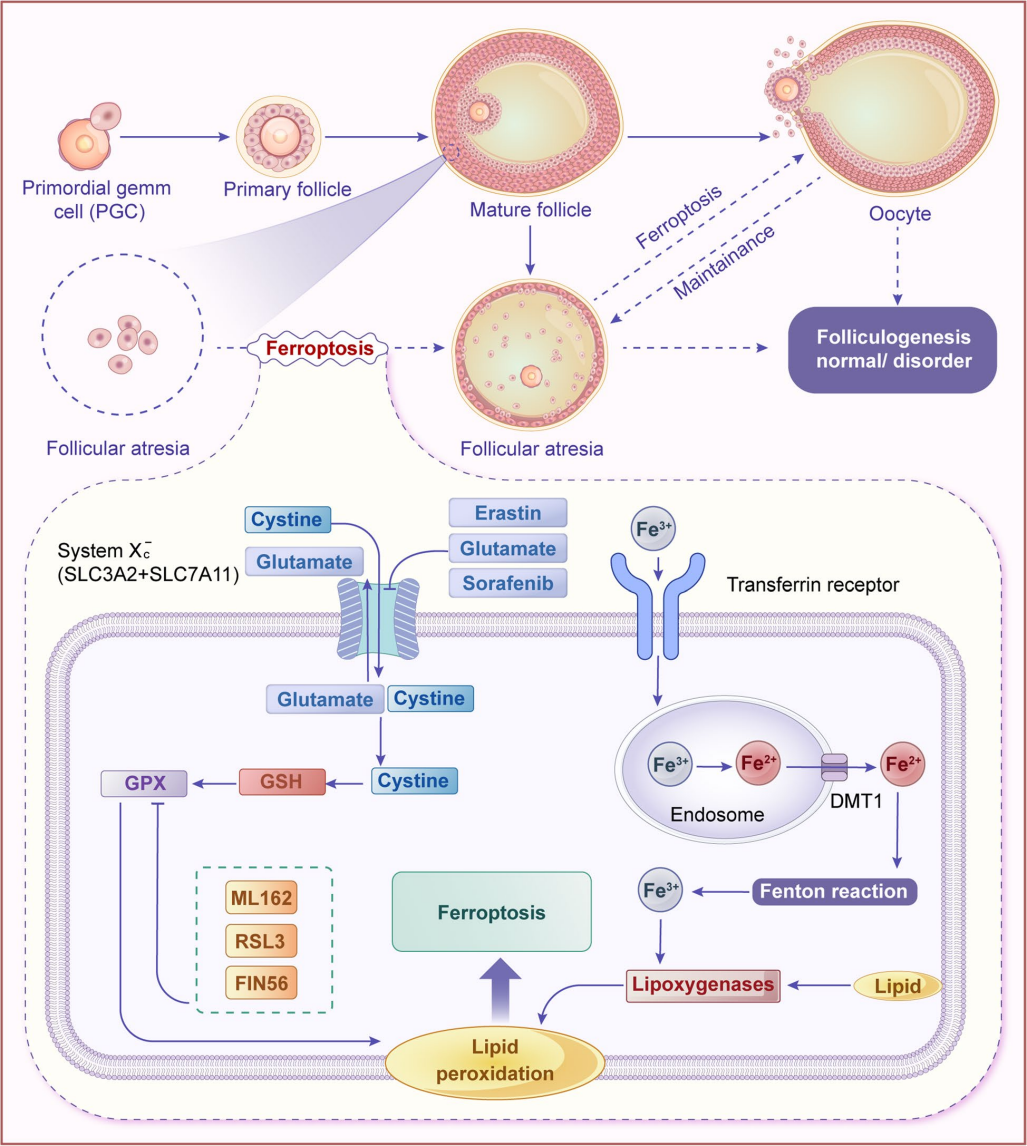
Ferroptosis as a regulator of follicular turnover. During follicular development, moderate ferroptosis eliminates dysfunctional cells, maintaining follicular pool health. However, excessive ferroptosis accelerates atresia by inducing granulosa cell and oocyte loss, impairing follicular growth. Elevated iron during atresia drives lipid peroxidation via the Fenton reaction, damaging membranes and activating ferroptosis pathways. Regulatory factors modulate ferroptosis to balance development and atresia, ensuring reproductive function. Ferroptosis thus functions as both a cellular quality control mechanism and a driver of pathological atresia

Preliminary evidence suggests that ferroptosis might directly damage follicular cells, potentially exacerbating developmental defects and contributing to oocyte quality decline [163]. Follicular atresia, a critical process in ovarian homeostasis, has been proposed to be regulated by ferroptosis (Fig. 6). Current mechanistic studies, primarily based on in vitro or rodent models, indicate that BNC1 deficiency could trigger ferroptosis through the NF2-YAP signaling pathway, resulting in aberrant lipid metabolism and primary ovarian insufficiency [164]. Iron-dependent oxidative stress further appears to promote atresia by inducing oocyte apoptosis [165]. These findings collectively establish ferroptosis as a central regulator of follicular atresia, particularly in ovarian dysfunction disorders like PCOS, where its activation drives follicular cell death and subsequent atresia [166]. While these findings are compelling, their generalizability to livestock physiology remains speculative due to the lack of direct in vivo evidence.

### Ferroptosis-related genes and their expression in follicular atresia

During follicular atresia, dysregulation of ferroptosis-related genes critically drives GC death and follicular degeneration. Heme oxygenase-1 (HMOX1) is upregulated under stress, promoting intracellular Fe^2^^+^ accumulation and MDA elevation, which triggers ferroptosis. Knockdown of *HMOX1* reduces Fe^2^^+^, ROS, and lipid peroxidation [167, 168]. Similarly, glutathione peroxidase 4 (*GPX4*), a key antioxidant enzyme, suppresses lipid peroxidation via glutathione (GSH)-dependent detoxification; its deficiency accelerates ferroptosis and follicular atresia [169–171]. Ferroptosis suppressor protein 1 (FSP1) mitigates oxidative damage, while its dysregulation disrupts iron homeostasis [172–174]. The cystine/glutamate antiporter* SLC7A11* maintains redox balance by supporting GSH synthesis, and its suppression enhances ferroptosis susceptibility [175]. *NRF2*, a master antioxidant regulator, transcriptionally activates SLC7A11 and GPX4, counteracting oxidative stress and ferroptosis [176–178]. These genes collectively regulate iron metabolism, lipid peroxidation, and antioxidant defenses, thereby influencing cellular fate and playing a pivotal role in modulating follicular development and atresia (Table 2).

**Table 2:** Expression profiles of ferroptosis-related genes in follicular atresia

Gene/Protein	Function	Role in atresia	References
HMOX1	Catalyzes heme degradation, releases Fe^2^^+^	Upregulated; promotes Fe^2+^ accumulation	[167, 168]
GPX4	Reduces lipid peroxides	Downregulated; permits LPO propagation	[169–171]
FSP1	Regulates CoQ10-NAD(P)H axis	Reduced expression enhances ferroptosis	[172–174]
SLC7A11	Cystine/glutamate antiporter; GSH synthesis	p53-mediated suppression induces ferroptosis	[175]
NRF2	Master antioxidant transcription factor	Activates GPX4 and SLC7A11 to inhibit LPO	[176–178]

### Iron homeostasis and oxidative stress in follicular atresia

Oxidative stress, fueled by iron overload, plays a pivotal role in triggering ferroptosis during follicular atresia. Excessive Fe^2+^ catalyzes the Fenton reaction, generating hydroxyl radicals (·OH) that initiate lipid peroxidation and membrane damage [179]. In poultry models, elevated iron levels in follicular fluid correlate with increased ROS production and granulosa cell ferroptosis [162]. Damaged mitochondria release excess ROS, further exacerbating oxidative stress. Ferroptotic cells exhibit characteristic mitochondrial changes, including increased membrane density and reduced cristae structure [180]. Heat stress induces mitochondrial ROS overproduction in GCs, leading to lipid peroxidation and follicular atresia [9, 160]. External factors such as heat stress, hypoxia, and environmental toxins amplify oxidative stress in ovarian follicles [111, 160]. Metabolic disorders like PCOS are associated with elevated intraovarian ROS levels and impaired antioxidant defenses [181, 182]. PUFAs in cell membranes are primary targets of ROS. Enzymes such as ACSL4 and LOX catalyze the peroxidation of PUFAs, leading to membrane destabilization and cell death [183, 184]. In geese, elevated MDA levels (a lipid peroxidation marker) in atretic follicles indicate ferroptosis activation [52]. The GPX4-GSH axis is the primary defense against lipid peroxidation. *GPX4* reduces lipid hydroperoxides to non-toxic alcohols, but its activity depends on GSH availability [47, 185]. Dysregulation of the cystine/glutamate antiporter (SLC7A11) depletes GSH, rendering cells vulnerable to ferroptosis [175]. *NRF2*, a master regulator of antioxidant responses, upregulates GPX4 and SLC7A11 to counteract oxidative stress [176–178]. *NRF2* activation in bovine GCs reduces ROS levels and inhibits ferroptosis, improving follicular survival [177]. Oxidative stress induces granulosa cell ferroptosis, disrupting follicular structure and hormone production, this impairs oocyte maturation and ovulation [162, 181]. In PCOS patients, granulosa cell ferroptosis is associated with reduced estradiol synthesis and impaired follicular development [182]. Heat stress-induced ferroptosis in porcine oocytes is associated with increased aneuploidy and developmental arrest, highlighting the detrimental effects of oxidative stress on reproductive outcomes [120]. These indicate that oxidative stress serves as a pivotal driver of ferroptosis during follicular atresia. While ferroptosis has been implicated in follicular atresia through in vitro studies and observations of iron overload and oxidative stress in granulosa cells, direct in vivo evidence in livestock ovaries remains limited. Further studies are warranted to confirm the role of ferroptosis in ovarian physiology across species.

## Emerging insights from single-cell studies

Recent advances in single-cell RNA sequencing (scRNA-seq) have provided unprecedented resolution in elucidating the cellular heterogeneity and molecular dynamics during follicular development and atresia. For instance, Li et al. delineated the transcriptional profiles of mouse follicular somatic cells, revealing stage-specific gene expression patterns that govern follicular selection and atresia [186]. Similarly, Zhang et al. identified key regulatory networks in chicken granulosa cells, highlighting autophagy and apoptosis pathways as critical determinants of follicular fate [187]. Zhao et al. further expanded these insights by mapping the trajectory of ovary aging, demonstrating how follicular somatic cells undergo progressive functional decline through altered metabolic and stress-response pathways [188]. These studies collectively underscore the utility of scRNA-seq in uncovering novel PCD-related mechanisms and cell-type-specific responses during follicular turnover. Future research should leverage such high-resolution approaches to dissect spatiotemporal PCD regulation across livestock species.

## Conclusions and perspectives

PCD plays a critical role in follicular development and atresia in livestock. These PCD pathways not only regulate follicular homeostasis but also exhibit complex interactions that determine follicular fate. For instance, autophagy can inhibit apoptosis by clearing damaged mitochondria, but under specific conditions, it may also promote cell death by activating apoptotic signaling pathways. Similarly, ferroptosis, driven by iron-dependent lipid peroxidation, interacts with autophagy and apoptosis, particularly under oxidative stress, to influence follicular survival or degeneration. These interactions highlight the intricate molecular network through which PCD pathways collectively regulate follicular development and atresia.

Future research should focus on elucidating the spatiotemporal regulatory mechanisms of PCD during follicular development, particularly by integrating single-cell sequencing and spatial transcriptomics to uncover the dynamic changes in PCD across different follicular stages. For instance, CRISPR-based screens could identify novel PCD regulators (e.g., GPX4 for ferroptosis or Bcl-2 for apoptosis) that enhance follicular resilience under stress conditions like heat or nutritional deprivation. Additionally, in-depth investigations into the interactions between PCD and reproductive hormones (e.g., FSH, LH, E2, and P4) will help reveal the molecular mechanisms underlying the hormone-PCD axis in follicular development and atresia. Practical applications could include dietary supplementation with antioxidants (e.g., vitamin E) or iron chelators to mitigate oxidative stress-induced ferroptosis in granulosa cells, as demonstrated in poultry models with improved egg production. Furthermore, dysregulation of PCD is closely associated with ovarian diseases such as PCOS and premature ovarian failure (POF). Translational studies using small-molecule inhibitors (e.g., ferrostatin-1 to block ferroptosis or rapamycin to induce protective autophagy) in livestock ovaries could pave the way for therapeutic interventions. Future studies should explore the role of PCD in these diseases to provide new insights for diagnosis and treatment. Finally, the development of molecular breeding strategies and reproductive regulation technologies based on PCD modulation holds promise for improving livestock reproductive efficiency. For example, selecting for genetic variants in PCD-related genes (e.g., *ATG5* or *SLC7A11*) through marker-assisted breeding could enhance follicular survival and litter size in pigs and cattle. Future research, leveraging advanced multi-omics, gene editing, and animal models, is poised to unlock breakthroughs in follicular development and livestock reproduction, paving the way for transformative advances in reproductive biology and breeding. While the core mechanisms of PCD are likely conserved across species, future studies should prioritize validating these pathways in livestock models to elucidate potential differences in signal responsiveness and hormonal regulation. Such efforts will enhance the translational relevance of these findings for improving reproductive efficiency in livestock.

## Data Availability

Not applicable.

## Abbreviations

AMH: Anti-Müllerian hormone
ATG5: Autophagy-related 5
ATG7: Autophagy-related 7
Cyt c: Cytochrome c
E2: Estradiol
FSH: Follicle stimulating Hormone
GCs: Granulosa cells
GPX4: Glutathione peroxidase 4
GSH: Glutathione
GnRH: Gonadotropin-releasing hormone
HMOX1: Heme oxygenase-1
LH: Luteinizing hormone
MDA: Reactive oxygen species
mTOR: Mammalian target of rapamycin
PCOS: Polycystic ovary syndrome
PCD: Programmed cell death
P4: Progesterone
PUFAs: Polyunsaturated fatty acids
ROS: Reactive oxygen species
SLC7A11: Cystine/glutamate antiporter
